# Synthesis and Properties of Thermotropic Copolyesters Based on Poly(ethylene terephthalate) and 4′-Acetoxy-4-biphenyl-carboxylic Acid

**DOI:** 10.3390/polym13111720

**Published:** 2021-05-24

**Authors:** Pavel A. Mikhaylov, Kirill V. Zuev, Marina P. Filatova, Boris Kh. Strelets, Valery G. Kulichikhin

**Affiliations:** A. V. Topchiev Institute of Petrochemical Synthesis, Russian Academy of Sciences (TIPS RAS), 29 Leninsky Prospekt, 119991 Moscow, Russia; pmih@ips.ac.ru (P.A.M.); zuev@ips.ac.ru (K.V.Z.); filatova@ips.ac.ru (M.P.F.); hammer41@bk.ru (B.K.S.)

**Keywords:** thermotropic copolyesters, PET, melt polycondensation, thermal behavior, viscoelasticity, polarizing microscopy, liquid crystalline state

## Abstract

A series of novel copolyesters based on polyethylene terephthalate (PET) and 4′-hydroxy-biphenyl-4-carboxylic acid (HBCA) was obtained by melt polycondensation of *bis*(2-hydroxyethyl) terephthalate and 4′-acetoxybiphenyl-4-carboxylic acid (ABCA) as co-monomers with Sb_2_O_3_ as a catalyst. Using this synthetic procedure, a set of copolymers containing 20–80 mol% of HBCA units was prepared. According to NMR spectroscopy, the copolymers were of random composition. Copolyesters comprising 60–80 mol% of HBCA possessed increased heat resistance and formed nematic melts at 270 °C and higher. The liquid crystal (LC) phase formation was accompanied by transition to non-Newtonian characteristics of the melt flow, as well as an equalization of storage and loss moduli values. According to XRD and polarizing microscopy, the LC glassy phase of the copolyesters coexists with crystalline regions of poly-(4′-hydroxy-4-biphenylcarboxylate), non-melting up to 400 °C and above. The mechanical characteristics of these LC copolyesters showed similar or better values than those of well-known LC polymers. These novel copolyesters can be useful in obtaining heat-resistant materials with an ordered structure and, as a consequence, improved performance.

## 1. Introduction

Among the wide range of melt-processed polymers, polyethylene terephthalate (PET) is one of the most important large-scale polyesters due to a number of valuable characteristics: relative cheapness, excellent mechanical properties, chemical resistance, and so on [1]. However, PET processing can be difficult because of a high rate of crystallization and limited heat resistance characterized by a low glass transition point. The thermal and mechanical properties of PET can be improved by physical and chemical modifications: (1) mixing with other polymers and (2) copolymerization [2]. In the first case, mixing PET with other polyesters, for example, polytrimethylene terephthalate [3], polyethylene naphthalate [4], or bisphenol A polycarbonate [5], is not a completely physical process since their monomeric units are exchanged between macromolecules up to complete randomization. Such a type of interaction is also known as transesterification or ester–ester exchange, and resulting products are copolyesters rather than blends.

Copolymerization is the more commonly used method of PET modification. Generally, commercial PET represents a copolyester where terephthalic acid is partially replaced with isophthalic acid. This reduces the crystallization rate, degree of crystallinity, and melting point. A copolyester containing about 35 mol% of 1,4-cyclohexanedimethanol instead of ethylene glycol is available commercially as PETG. The main features of PETG are a very low crystallinity degree and reduced moisture absorption, so it can be processed without preliminary drying [6].

A number of PET-based copolyesters form a liquid crystal (LC) phase in the melt. Among them, a copolyester based on PET and 4-hydroxybenzoic acid (HBA) is the most studied and has found wide range of applications as an engineering plastic [7]. PET/HBA copolyesters containing 60–80 mol% of HBA are commercially available, for example, under the trademark Rodrun [8]. Compositional heterogeneity is inevitable for such copolymers, but this can be reduced by altering the synthetic procedure: preliminary acidolysis of PET with acetic acid and subsequent stepwise addition of HBA improves the statistical characteristics of copolyester macromolecules [9]. Additional known copolymers PET and 6-hydroxy-2-naphthoic acid (HNA) have been prepared in a similar way [10]. Some copolyesters containing 4,4′-substituted biphenyl units also form LC melts [11]. For example, melts of copolymers based on PET and polyethylene biphenyl-4,4′-dicarboxylate also exhibit birefringence [12,13,14]. It has been suggested that the solid-state structure of these copolymers contains crystalline and frozen liquid crystalline phases, so-called anisotropic glass [15]. Copolymers based on PET and containing 4,4′-biphenol terephthalate, forming LC melts, are also described [16]. The papers mentioned above refer to a modification method in which mesogenic fragments are introduced into the native polymer (PET).

Simultaneously, there exists a wide field of research activity in preparing LC copolyesters using mesogenic low molecular weight monomers. Among such monomers, 4′-hydroxybiphenyl-4-carboxylic acid (HBCA) deserves special attention. There is a known infusible homopolymer of HBCA that has an extremely high thermal stability, even exceeding that of poly-4-hydroxybenzoate [17]. However, few studies have examined copolyesters produced from HBCA. For example, the preparation of thermotropic fully aromatic copolyesters based on HBCA and HNA, as well as ternary copolymers of HBCA, HNA, and HBA, has been described [18]. Some aliphatic diols and ethyl esters of HBCA have been employed to obtain alkylene-aromatic main-chain thermotropic copolyesters in solution using the Mitsunobu reaction [19,20]. Attention was paid to the low rate of mesophase formation and specific relaxation behavior, and it was found that the glass transition temperatures of amorphous and LC phases were different. In these cases, the use of hydroxybiphenylcarboxylic acids instead of the traditional dicarboxylic acids and diols mixtures does not require the use of a diol, which is expected to increase the compositional homogeneity of the copolymers.

Nevertheless, we could not find any indication of using HBCA for PET modification, though it is reasonable to expect interesting properties of prepared copolyesters due to their rigid structure with high aspect ratio, inherent to HBCA mesogenic units. An attempt to combine PET and HBCA to prepare the novel thermotropic copolyester was the driving force of this research. The distinctive feature of this work is preparing PET-based copolyesters by melt polycondensation of 4′-acetoxybiphenyl-4-carboxylic acid (ABCA) and *bis*(2-hydroxyethyl) terephthalate (BHET). Earlier, it was shown by our group [10] that using acetoxy derivatives of HBA could diminish the formation of block sequences in PET/HBA copolyesters. Therefore, the preparation of a series of novel thermotropic copolyesters based on PET and ABCA as well as their structure and properties were the main focus of this research. For comparative analysis, HBCA homopolymer was also obtained and analyzed.

## 2. Materials and Methods

Materials: Most of the solvents (analytical grade) and ditolylmethane (reagent grade, a mixture of isomers with bp = 292–296 °C) were obtained from Ekos-1 LLC (Moscow district, Russia) and used without additional purification. Dichloroacetic acid (analytical grade) was purchased from Brie LLC (Nizhny Novgorod, Russia), pentafluorophenol (99% purity) from Fluorochem (Hadfield, UK), trifluoroacetic acid (98% purity) and trifluoromethanesulfonic acid (98% purity) from Fluka (Honeywell global delivery), chloroform-*d* (99.8% purity) and DMSO-*d*_6_ (99.8% purity) from Cambridge Isotope Laboratories (Tewksbury, MA, USA), 4-hydroxybenzoic acid (>99% purity) from *Acros Organics*, and 4′-acetoxybiphenyl-4-carboxylic acid (ABCA) from Yaroslavl Federal State University (Yaroslavl, Russia). The purity of ABCA was verified by thin layer chromatography on Silufol 254 nm plates and NMR spectroscopy in DMSO-*d*_6_ (^1^H NMR 300 MHz; *δ*, ppm: 8.01 (*d*, *J* = 8.1 Hz, 2H), 7.84–7.68 (*m*, 4H), 7.23 (*d*, *J* = 8.5 Hz, 2H), 2.27 (*s*, 3H)). Commercially produced PET (copolymer containing 0.2% polyethylene isophthalate, [*η*] = 0.83 dl/g in dichloroacetic acid at 25 °C) was provided by New Polymers Plant “Senege” (Moscow district, Russia).

Two well-proven thermotropic LC polymers Zenite HX-8000 (*Du Pont*) prepared from monomers (4-hydroxybenzoic, terephthalic acids, and hydroquinone derivatives [21] and PET/HBA (40/60 mol%) copolyester (Rodrun analog) synthesized from polymer/monomer mixture) were used as reference samples for comparative mechanical tests. The most commonly used processing temperature of Zenite HX-8000 is 270–290 °C. Processing temperature of PET/HBA copolyester was about 220–250 °C. The choice of these copolyesters as reference samples was due to the following. The PET/HBA copolymer and polyesters obtained in this work are the closest analogs both in structure (both are alkylene-aromatic main-chain TLCP) as well as in the synthesis method (based on native PET). The only difference is the aspect ratio of the mesogenic fragments. Zenite HX-8000 is a fully aromatic copolyester and contains similar structural units. The mechanical characteristics of fully aromatic polyesters are generally superior to those of alkylene-aromatic ones, which is why the HX-8000 was chosen as the “high standard” sample.

Polymer synthesis: The melt polycondensation process was carried out in 100 or 250 mL three-necked flasks equipped with a mechanical stirrer, inlet for inert gas, and outlet to an oil vacuum pump. A detailed synthesis procedure was described earlier [10]. The reaction flask, loaded with PET, was evacuated and then filled with argon (three times) and immersed in a metal bath heated to 270–280 °C. After complete PET melting, ethylene glycol (EG) was added dropwise to the reaction flask until the addition of a new EG portion did not cause boiling of the mixture when the metal bath was cooled to 230–240 °C and 200–250 ppm of catalyst (Sb_2_O_3_) was added. Then, ABCA was added, and the temperature increased to 280–295 °C. Acetic acid was released for 30–40 min under a slow stream of argon, and the polycondensation continued under vacuum, gradually reducing the pressure to ~0.5 mm Hg. When the required mixture viscosity was reached (visual control), the flask was filled with argon and the polymer discharged onto a polyimide film and cooled. This method was used to obtain copolymers with initial molar ABCA concentrations of 20% (C20), 40% (C40), 60% (C60), and 80% (C80) relative to one monomer unit of PET.

PET/HBA (40/60 mol%) copolyester was prepared by melt transesterification in a 100 mL three-neck round-bottomed flask equipped with glass paddle mechanical stirrer, nitrogen inlet, and vacuum outlet according to the previously described method [7]. Briefly, 10 g (52 mmol) of PET and 14 g of ABA (78 mmol) were dried in vacuum for about 30 min at 120 °C in the reaction flask and placed into a preheated metal bath. The flask was then filled with nitrogen and evacuated three times. The temperature of the metal bath was rapidly increased to 275 °C, maintaining a slow stream of nitrogen. The mixture melted providing a clear homogeneous melt, and acetic acid began to form. After 30–40 min of stirring, a vacuum was applied for 4 h. Finally, the white opaque product was discharged onto polyimide film and cooled. Intrinsic viscosity [*η*] = 0.73 dL/g (in dichloroacetic acid at 25 °C). The copolymer composition was confirmed by ^1^H NMR spectroscopy.

Homopolymer HBCA (B100) was prepared by a procedure similar to that previously described [18,22]. A 100 mL three-necked flask equipped with a mechanical stirrer was charged with 5 g of ABCA and 25 mL of ditolylmethane. The flask was heated to 220 °C in a metal bath while argon was continuously bubbled through the reaction mixture. The temperature was raised to 300–310 °C for 1 h and maintained for 16 h. After cooling the mixture, the polymer was collected on a filter, washed with hot acetone for 24 h in a Soxhlet extractor, and dried.

Copolyester analysis: The measurement of intrinsic viscosity for copolyesters C20, C40, C60, and PET/HBA was carried out at 25 ± 0.1 °C in dichloroacetic acid (DCA) using an Ubbelohde viscometer in accordance with ISO 1628-5-1998. Copolyester C80 is insoluble in DCA, so the logarithmic reduced viscosity in pentafluorophenol at 60 ± 0.1 °C was determined using the formula: η* = ln (*t*/*t*_0_)/*c*, where (*t*/*t*_0_) is the ratio of the 0.1% polymer solution flow time to the solvent flow time.

High-resolution ^1^H NMR spectra for ABCA (in DMSO-*d*_6_), PET (in CDCl_3_/CF_3_COOH mixture, 5:2 by vol.), and C80 (in CF_3_COOH/CDCl_3_/CF_3_SO_3_H mixture, 1:2:1 by vol.) were recorded on an MSL-300 spectrometer (Bruker, Billerica, MA, USA) with an operating frequency of 300 MHz (frequency sweep 8928 Hz, 90° pulse 3 μs, sampling time 1.835 s, 20 accumulations, pulse program PAPS.PC with subsequent Fourier transform, temperature 24 °C). Chemical shifts were calculated from the signal of residual protons of solvent (chloroform 7.26 ppm or DMSO 2.50 ppm). ^1^H NMR spectra for PET/HBA, C20, C40, and C60 (in CDCl_3_/CF_3_COOH mixture, 5:2 by vol.) were recorded on an Avance spectrometer (Bruker, Billerica, MA, USA) with an operating frequency of 700 MHz. Chemical shift of residual chloroform protons was used as reference.

FTIR spectra were recorded in the reflection mode on a Hyperion-2000 IR-microscope coupled to an IFS-66 v/s IR-Fourier spectrometer (Bruker, Billerica, MA, USA): crystal Ge, resolution 2 cm^−1^, wavelength range 4000–600 cm^−1^).

Thermal behavior. DSC thermograms of copolyesters were measured and analyzed using two instruments—MDSC 2920 (TA Instruments, New Castle, DE, USA) and DSC 3+ (Mettler-Toledo, Columbus, OH, USA)—in the following mode: heating to 450 °C and cooling to 20 °C at 20 K/min in an inert atmosphere (argon). Thermogravimetric analysis (TGA) was performed on a Derivatograph-1500Q instrument (*MOM*, Hungary) in the heating mode up to 1100 °C at 10 K/min in air.

X-ray diffraction analysis was performed using a rotating copper anode Rotaflex RU-200 (Rigaku, Tokyo, Japan) in 50 kV–100 mA source operating mode. The X-ray source was equipped with a horizontal wide-angle D/Max-RC goniometer and a secondary graphite monochromator (emission wavelength *λ* = 1.542 Å). The range of diffraction angles was 5–60° in 2*θ*, and measurements were carried out in the continuous scanning mode at a rate of 2°/min (step 0.04°). *θ*–2*θ* scanning was performed according to the Bragg–Brentano scheme.

The rheological characteristics of copolyester melts were measured at 270 °C using a Haake Rheostress 600 rotational rheometer (Thermo Fisher Scientific, Waltham, MA, USA) in the stationary shear deformation (shear rate range 0.01–1000 s^−1^) and oscillation (amplitude range 0.1–1000 Pa, frequencies range 1–100 Hz) modes using a cone-and-plate operating unit (diameter 20 mm, apex angle 1°). The measurements were carried out in air within no more than 15 min per experiment. Copolyester samples were dried at 160 °C for 24 h prior to the measurements.

The optical properties of thin films of copolyester melts were studied using a 6 PO polarizing optical microscope (Biomed, Moscow, Russia) equipped with a FP900 heating table (Mettler, Columbus, OH, USA) and an E3ISPM5000 photo/video camera (ToupTek Photonics Co., Zhejiang, China), in the following mode: heating to 375 °C and cooling to 25 °C (20 K/min). An inert atmosphere was maintained in the heating cell.

Mechanical testing. Copolyester films were prepared by compression molding at 220 (C20–C60, PET), 250 (C80), 230 (PET/HBA), and 270 °C (Zenite HX-8000). The mechanical properties of the films were measured on an Instron 1122 tensile testing machine (UK) with a base length of 10 mm at a tensile speed of 10 mm/min.

## 3. Results and Discussion

### 3.1. Synthesis and Structure of Copolyesters

**Scheme 1:**
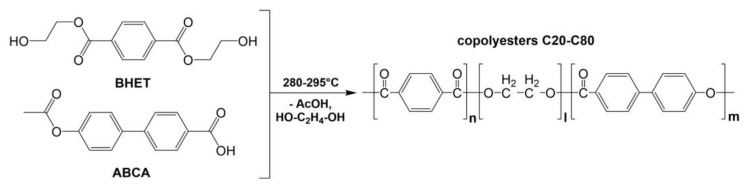
The route for the synthesis of copolyesters C20–C80.

The route for the synthesis of PET/HBCA copolyesters is shown in Scheme 1. The starting monomers were 4′-acetoxybiphenyl-4-carboxylic acid (ABCA) and *bis*(2-hydroxyethyl) terephthalate (BHET) obtained by glycolysis of PET with an excess of EG. After near-complete glycolysis of PET, the catalyst and the second monomer (ABCA) were added. Initial stage of synthesis was performed in an inert atmosphere, while the main byproduct was acetic acid (AcOH). Further polycondensation was continued in a vacuum to more efficiently remove the released AcOH and EG.

According to this scheme, a number of copolyesters with HBCA content from 20 to 80 mol% relative to PET monomer unit were obtained. The duration of the vacuum stage of syntheses, copolyester intrinsic viscosity, and the monomer ratio are given in Table 1. It should be noted that two polycondensation byproducts were formed during the syntheses: AcOH (bp = 118 °C), which is easily removed from the reaction mixture, and EG (bp = 197 °C). Increased ABCA content in the reaction mixture results in shortening the vacuum stage of polycondensation, which may be explained by the decreased amount of BHET, which gives a less volatile polycondensation byproduct (EG) than ABCA (AcOH).

**Figure 1:**
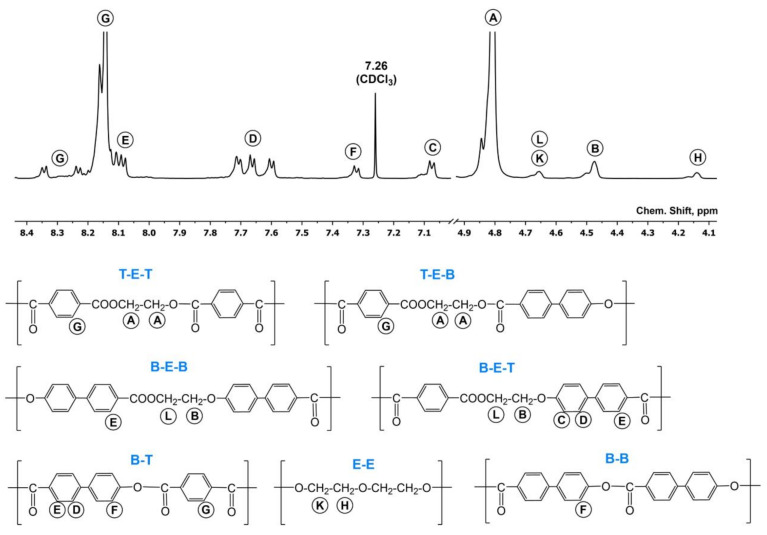
The ^1^H NMR spectrum of copolyester C20 and possible structural units (dyads and triads) of C20–C80 copolyesters. The protons detected by ^1^H NMR are marked by letters A–G.

^1^H NMR was used to analyze the composition of copolymer macromolecules. As an example, Figure 1 shows the spectrum of C20 copolyester. Similar, but slightly broader signals were observed in the NMR spectra of C40–C80 copolymers. The possible sequences of structural units for these copolyesters are also shown in Figure 1. The assignment of spectra signals was made in accordance with calculated intensities and chemical shifts of signals, spin–spin coupling constants, analysis of previous work [23,24,25], and analysis of the NMR spectrum of the HBCA homopolymer. The corresponding results are presented in Table 2.

Three groups of signals from aliphatic protons are observed in the spectra. In the region of 4.75–4.89 ppm, there are overlapped EG signals belonging to the B–E–B, B–E–T, and T–E–T triads, as well as signals of the B–E dyads from methylene groups linked to carboxyl groups by an ester bond. Signals from diethylene glycol (DEG) units E–E lie in the regions of 4.64–4.70 and 4.12–4.19 ppm. Signals from methylene groups of Ar–O–CH_2_– fragments (B–E dyads) are located in the range 4.45–4.53 ppm. In the aromatic region of the spectra there are three signals from proton C of the B–E dyad (7.05–7.13 ppm) and two signals from proton F of B–T dyad (7.30–7.38 ppm). The intensity of the D signal group is twice that of the F + C signals, which proves their correct assignment. In the region 8.06–8.37 ppm, signals from protons of terephthalic acid (TPA) in various triads appear, as well as signals from E-protons in the *o*-position to the carboxyl groups of HBCA units. Since C80 is insoluble in CF_3_COOH, its spectrum was obtained by dissolving it in a CF_3_SO_3_H/CF_3_COOH/CDCl_3_ mixture. That is why the aliphatic part of the spectrum turned out to be highly distorted and was not considered.

Based on the assignment, the molar ratio of HBCA/(HBCA + TPA) (*N*_B_), the proportion of DEG residues in relation to the total number of EG units (*N*_EE_/*N*_E_), the percentage of HBCA in the sequences T–E–**B**–E (*N*_TBE_/*N*_B_), B–**B**–E–T(B) (*N*_BBE_/*N*_B_), T–E–**B**–T (*N*_TBT_/*N*_B_), and B–**B**–B (*N*_BBT_/*N*_B_) in copolymers C20–C60 were determined (Table 3).

The signals in the ^1^H NMR spectrum of the C80 copolymer were found to be strongly broadened. Therefore, for C80 the *N*_B_ values, the [*N*_TBE_/*N*_B_ + *N*_BBE_/*N*_B_] sum and the [*N*_TBT_/*N*_B_ + *N*_BBT_/*N*_B_] sum were estimated. The obtained results show that samples C20–C60 are random copolymers. The ratio of TPA to HBCA units in the C20–C80 copolymers corresponds practically to the initial loading of monomers, but the content of EG/DEG residues is higher than twice the sum of carboxyl residues due to the presence of B–E–T fragments. In addition, the high content of DEG fragments in the C20 copolymer (3.1%) should be noted.

**Figure 2:**
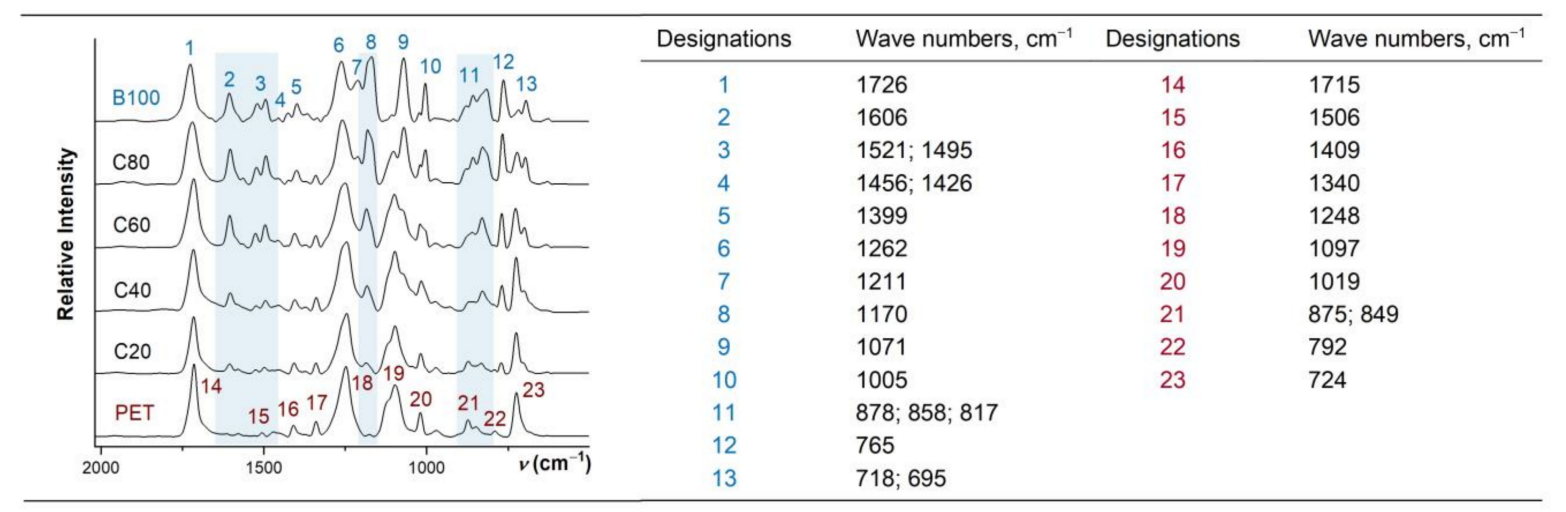
FTIR spectra of PET, homopolymer B100, and copolyesters C20–C80. The main signals decoded in the spectra of PET and B100 are also shown.

A series of absorption bands characterizing the *p*-substituted aromatic ring (1606 and ~1500 cm^−1^, several *δ*_CCH_ signals in the region of 850–730 cm^−1^) were observed in the FTIR spectrum of homopolymer B100 (Figure 2). In the range of 1300 to 1000 cm^−1^ the bands *ν*_CCH_ and *ν*_C–O_ (1211 and 1170 cm^−1^) were found. These bands were also detected in the spectra of C80–C20 copolyesters, and their intensity decreased with lowered HBCA content. The position of the carbonyl group *ν*_C=O_ band shifted toward higher frequencies with an increase in the HBCA content: from 1715 (PET) to 1726 cm^−1^ (B100). This occurred due to the shortening of the C=O bond caused by the electron effect from aromatic ring.

**Figure 3:**
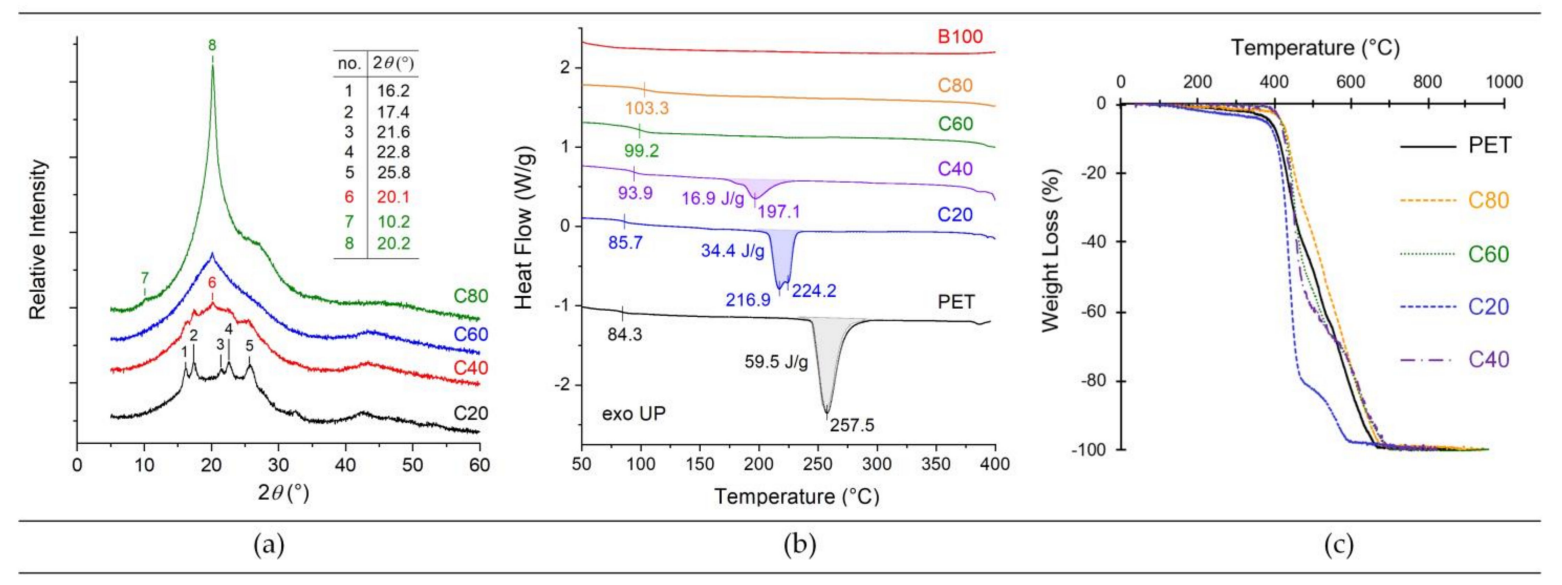
(**a**) XRD patterns of copolyesters C20–C80. (**b**) DSC thermograms for PET, copolyesters C20–C80, and homopolymer HBCA (B100) in the first heating cycle at a rate of 20 K/min in an inert atmosphere. (**c**) TGA data for PET and C20–C80 copolyesters in air at a heating rate of 10 K/min.

The XRD patterns of C20–C40 copolymers (Figure 3a) show characteristic reflections inherent to PET and similar PET/HBA copolymers with an HBA content less than 40 mol% [26,27]. The 2*θ* diffraction maximum of ~20°, corresponding to the crystalline structure of the HBCA homopolymer [18,22], appears in the diffractograms beginning with C40, and its intensity increases with increasing HBCA content. In the C40–C80 series, a slight shift in the position of this diffraction maximum is observed: 20.1° for C40 and 20.20° for C80. The crystalline phase of PET disappears almost completely for C60–C80 copolyesters, and crystalline phase of HBCA prevails. In the region of 5–10° a broad low-intensity peak of ~10° is observed only for copolyester C80, which can be explained by the presence of glassed mesophase region [15,19].

### 3.2. Thermal Properties of Copolyesters

The melting temperatures and enthalpies, as well as glass transition temperatures (*T*_g_) for PET, C20–C80 copolyesters, and HBCA homopolymer, are shown in Figure 3b. The results of DSC analysis are given in Table 4. An increase in the content of HBCA units in copolymers resulted in decreasing melting and crystallization enthalpies and a significant increase in the glass transition temperature. This behavior of *T*_g_ is contradictory to that previously described for PET/HBA copolyesters, which do not demonstrate a noticeable increase of *T*_g_ up to 63 mol% HBA [28].

A rather low glass transition temperature (*T*_g_ ~ 100 °C) is also observed in thermotropic Vectra A copolymers [11,29]. On the other hand, the introduction of diphenyl co-monomers increases the glass transition point for the copolymer of polyhydroxybenzoic acid (67%) and 4,4′-biphenol terephthalate (33%) to 180 °C [30], although this is a fully aromatic copolyester. Recently, an increased *T*_g_ value (up to 113 °C) was revealed for ternary DEG, 4,4′-biphenylydicarboxylic acid, and 3,4′-biphenylydicarboxylic acid copolyesters [31].

The thermograms for C60–C80 copolymers and B100 homopolymer do not demonstrate endo- or exothermic effects, which indicates either their amorphous or mesophase structure or lack of phase transitions in the temperature range of 50 to 400 °C. At the same time, for copolymers C20 and C40 there was a noticeable decrease in melting temperatures compared to PET, apparently due to the randomization effect. Such randomization, leading to depression of the crystalline phase as a result of the introduction of new units into PET, has been repeatedly mentioned in the literature. For example, copolyesters of PET and 2,6-hydroxy naphthoic acid (HNA) lose crystallinity even when the content of HNA is about 10 mol% [10]. The same effect has been observed in the analysis of so-called PETG copolymers with different contents of 1,3- and 1,4-cyclohexanedimethanol (CHDM): Amorphization occurs when the content of CHDM units is over 30 mol%, and as their content increases, the glass transition temperature increases, and the melting temperature of copolyesters decreases [32]. Nevertheless, according to XRD data, the crystalline phase exists in in solid samples C60 and C80, but its melting point is higher than 400 °C.

Thermogravimetric analysis shows (Figure 3c, Table 5) that with increased HBCA content in copolyesters the onset temperature of weight loss increases, which indicates an increase in thermal stability from C20 to C80. A similar effect was observed for copolyesters PET/HBA [26,33] and PBT/HBA [34]. It is interesting to note that the corresponding temperatures of weight loss, inherent to PET, are higher than for C20. Presumably, this effect is also explained by randomization of the copolyester chains. Possible explanations of the poor thermal stability of C20 are the relatively high DEG content and large number of unstable ether bonds. The role of these factors in the degradation of PET has been well studied: The ether links are first affected by the thermal oxidation, forming radicals and further quinone-type structures in the PET backbone; in addition, DEG blocks are reactive sites where degradation can start with the formation of intramolecular structures [2,24].

### 3.3. Melt Rheology

**Figure 4:**
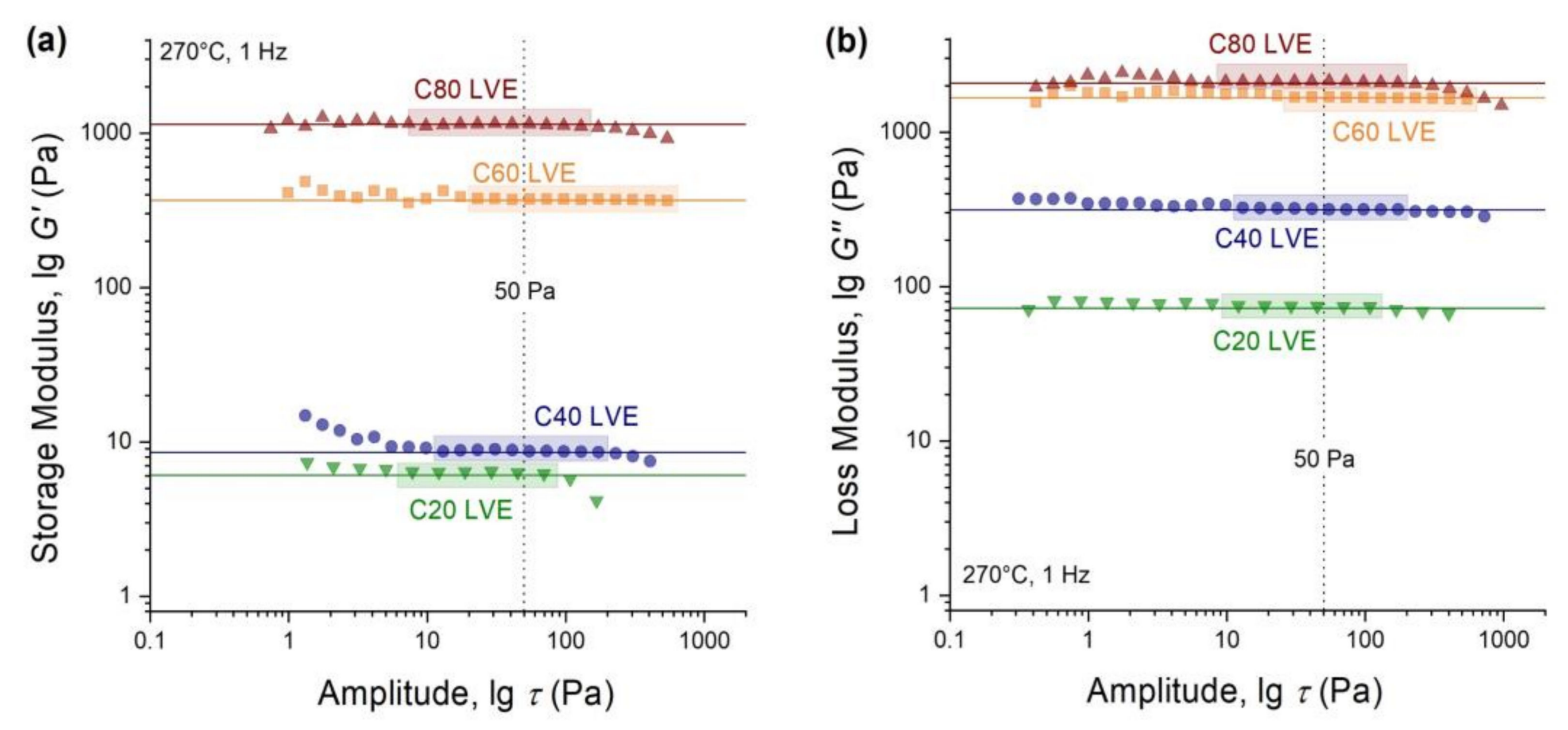
Dependence of the storage modulus (**a**) and loss modulus (**b**) on the stress amplitude for C20–C80 copolyester melts at 270 °C.

Changes in the structure of copolyesters C20–C80 along the composition are reflected in their rheological characteristics. Figure 4 shows the dependence of the storage and loss moduli on the stress amplitude in oscillation mode with a frequency of 1 Hz for C20–C80 melts at 270 °C. With increased HBCA content, a significant increase in both viscous (G″) and elastic (G′) components of the complex modulus for copolyesters melts is observed. For all samples in the entire amplitude range, the G″ values are higher than G′. Switching from C20 to C80, the difference between the G″ and G′ values decreases. Thus, the higher the content of biphenyl units in macromolecules, the lower the mechanical loss tangent values: tan *δ* >> 20 for C20, 10–20 for C40, 2–5 for C60, and ~1 for C80, which means an increase in the melt’s elasticity occurs. Since the C60 and C80 copolyesters are rigid-chain, the tan *δ* → 1 indicates a high ordering of macromolecules and the presence of a domain flow [35]. The region of linear viscoelasticity (LVE) for all samples covers approximately the same stress range—up to 100 Pa.

**Figure 5:**
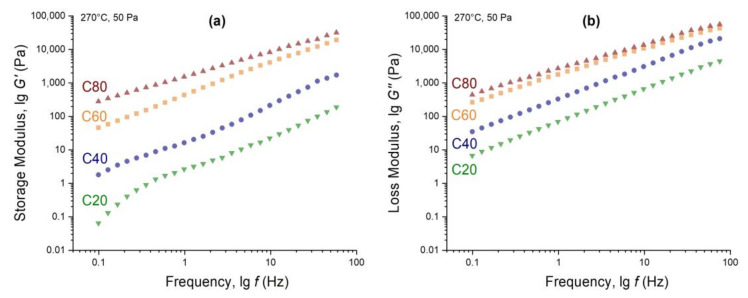
Dependence of the storage modulus (**a**) and loss modulus (**b**) on frequency with an amplitude of 50 Pa for C20–C80 copolyester melts at 270 °C.

The amplitude value of 50 Pa selected for all copolyesters in the LVE region was used to measure the frequency dependence of the complex modulus components in the range of 1 to 100 Hz, also at 270 °C (Figure 5). Viscous characteristics of melts prevail over elastic ones (*G″* > *G′*), similarly to the amplitude sweep, for all samples over the entire frequency range. Frequency dependence of the modules does not intersect, i.e., a crossover point was not detected. At 270 °C, the C20 copolyester melt is completely isotropic and has a very low viscosity. Therefore, in the region of low frequencies (up to 1 Hz), the processes of disordering due to thermal motion are predominant, and the value of tan *δ* takes on very high values—up to 200. A further increase in the oscillation intensity leads to a decrease in tan *δ* to ~20. The mechanical losses of other copolyesters change much less with varying frequency.

**Figure 6:**
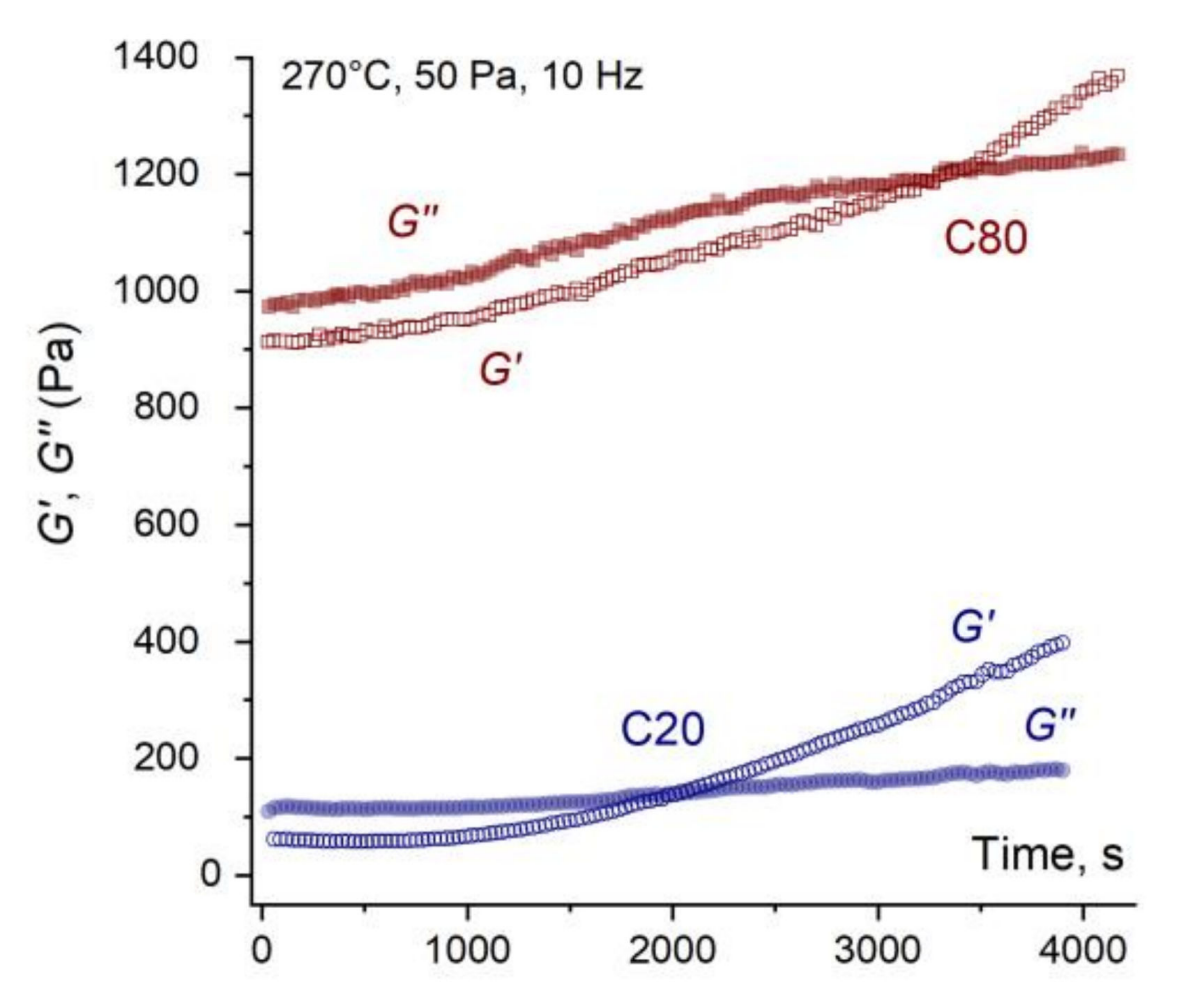
Evolution of the complex modulus components in time at strain of 50 Pa and frequency of 10 Hz for melts C20 and C80 at 270 °C.

Nevertheless, the crossover point can be reached in time during a sufficiently long period (over 15 min) of melt exposure. An increase in the viscous and elastic characteristics of copolyesters occurs over time, while *G′* increases faster than *G″*, and a transition to the region where G′ > G″ takes place within 1 h (Figure 6).

The most plausible reason for such evolution is changes of the composition of the copolyesters due to the continuation of chemical processes. According to ^1^H NMR data, the content of DEG units (–E–E– in Figure 1) in the C20 copolymer is higher than that of other copolymers of the series (Table 3). It has been shown [24] that diethylene glycol fragments, which are always present in PET, play an essential role in thermal oxidative degradation, which is also confirmed by TGA data for poly(diethylene glycol terephthalate) [25]. Usually, a decrease in polymer viscosity should be observed during its thermal decomposition, but in our case the moduli of C20 and C80 increased over time, which means that oxidation may initiate further chemical processes.

The most plausible reason for such evolution is changes of the composition of the copolyesters due to the continuation of chemical processes. According to ^1^H NMR data, the content of DEG units (–E–E– in Figure 1) in the C20 copolymer is higher than that of other copolymers of the series (Table 3). It has been shown [24] that diethylene glycol fragments, which are always present in PET, play an essential role in thermal oxidative degradation, which is also confirmed by TGA data for poly(diethylene glycol terephthalate) [25]. Usually, a decrease in polymer viscosity should be observed during its thermal decomposition, but in our case the moduli of C20 and C80 increased over time, which means that oxidation may initiate further chemical processes.

As seen in Figure 6, for copolymer C20 the crossover point is reached sooner. It is possible that the decomposition products of DEG fragments can catalyze a direct chemical reaction. This is supported by the results of thermogravimetric analysis (Table 5): Sample C80, which has a lower content of nascent PET compared to C20, has a higher thermal decomposition temperature, and the transition over crossover point occurs later. As previously reported [36], copolyesters of PET and 4-hydroxybenzoic acid were studied in an inert atmosphere, and such an increase in the complex dynamic modulus components was also observed. In our case, the increase in G′ was more significant, and the transition to the region where G′ > G″ occurred after 2.5 or 4 h for polyesters with a molar content of HBA units of 60 or 80%, respectively. According to these data, it is most likely that the effect was associated with continuation of the polycondensation reaction.

**Figure 7:**
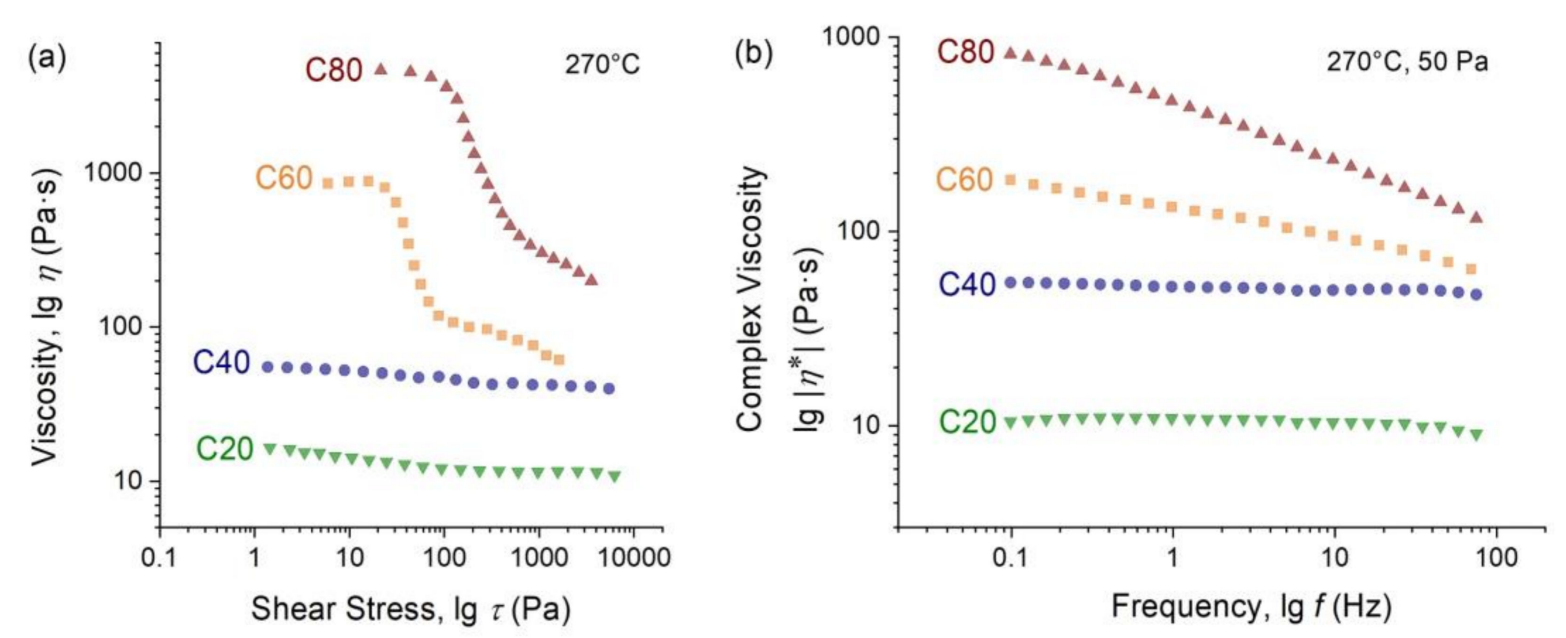
Flow curves of C20–C80 copolyester melts obtained at stationary shear (**a**) and oscillation (**b**) modes at 270 °C.

Flow curves obtained in stationary shear mode (range of shear rates: 0.01–1000 s^−1^) and the frequency dependence of complex viscosity (frequency range 1–100 Hz) for copolyester melts that did not undergo long exposure to air are presented in Figure 7. There are two general features of the rheological behavior with an increase in HBCA content from 20 to 80%: (1) a significant viscosity increase (up to two orders of magnitude) and (2) the appearance of a viscosity anomaly, especially noticeable when the content of diphenyl fragments is more than 60. The Cox–Merz rule is quite well fulfilled at equal shear rates and frequencies for C20 and C40 copolyesters, while the differences in viscosity values obtained in stationary and oscillatory modes are significant for C60 and C80 (Table 6).

Such behavior is not unusual for this type of polymer. A flow pattern similar to that of sample C80 was observed for copolyester PET/HBA (20/80 mol%) [37], including the existence of the G′ ≈ G″ region, and a transition to strong non-Newtonian up to yield-like behavior. This was explained by the presence of melt zones enriched with infusible crystallites of poly-HBA. It was also shown that PET/HBA copolymers with a low content of HBA form isotropic melts with a Newtonian flow, similar to samples C20–C40 [33]. With an increase in the content of HBA units above 60 mol%, an anisotropic melt forms with two regions in the flow curves: quasi-Newtonian at low deformation rates and pseudoplastic at high ones (as in the case of C60–C80 samples). Discrepancies in the values of *η* and |η*| for C60 and C80 melts could be explained by orientation effects at steady-state flow, which are inherent to nematic polymers.

Such a viscosity anomaly is often observed for melts of rigid-chain polymers upon transition to the liquid crystalline state [38]. An increase in the content of mesogenic units in macromolecules leads to a transformation of the flow curve shape. The final curve of thermotropic LC polymers usually has three characteristic flow zones: (1) viscoelastic behavior with a yield point caused by domain motion of the LC phase elements without significant orientation changes; (2) quasi-Newtonian flow of oriented LC domains; and (3) sharply non-Newtonian behavior due to fragmentation and reorientation of the LC phase domains [39,40]. In our case, only zones 2 and 3 were observed for copolyesters C60 and C80 (Figure 7a), which can be explained by insufficiently long conditioning of the melts at the measurement temperature in order to avoid thermal oxidative processes.

### 3.4. Polarizing Light Microscopy

**Figure 8:**
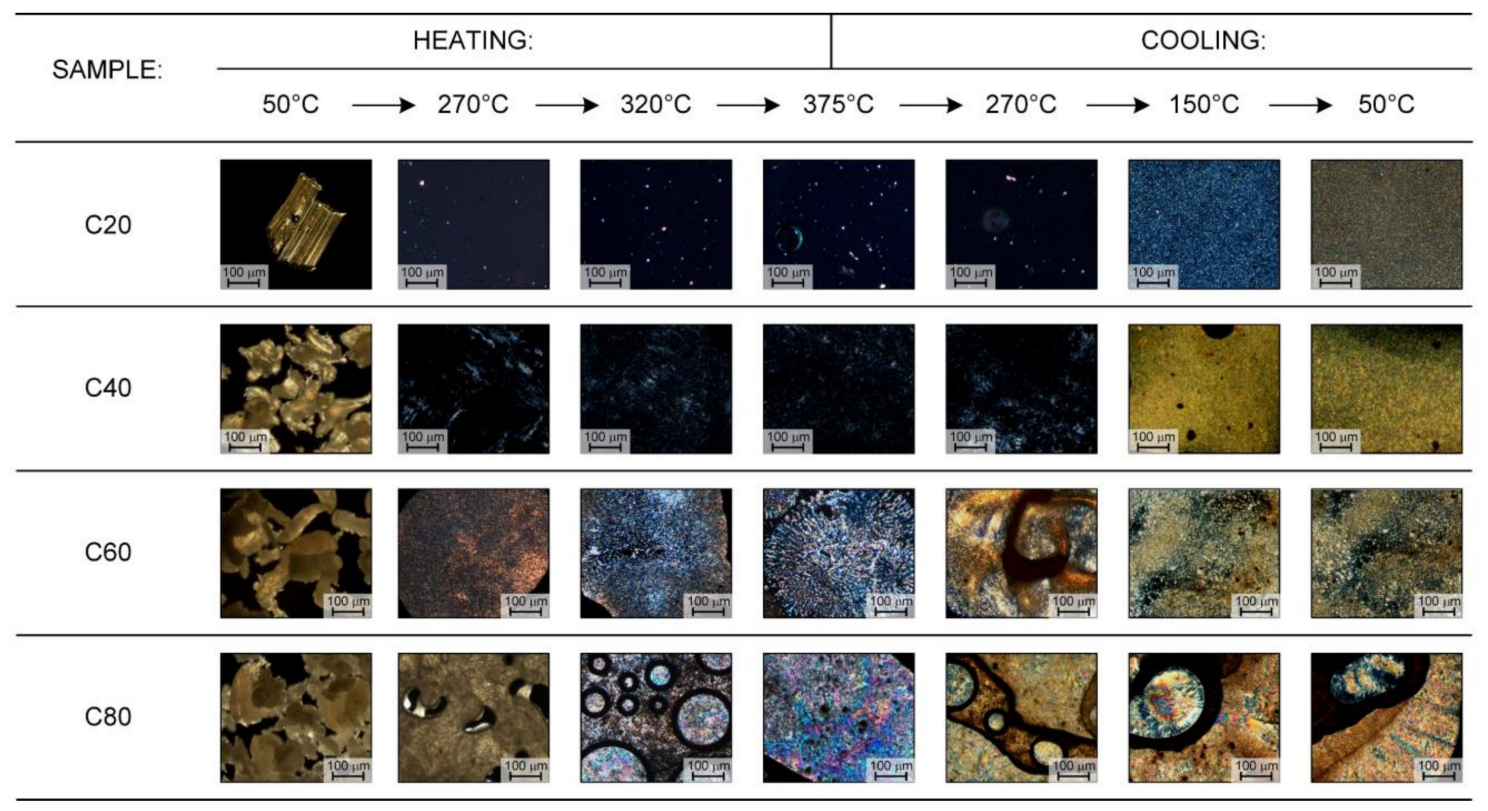
Micrographs of C20–C80 melts obtained in crossed polarizers during heating to 375 °C and subsequent cooling to 50 °C.

In order to observe the mesophase formation, copolyester melts was analyzed by polarizing microscopy. Polyester samples (pellets), placed between the slide and cover glasses, were sequentially heated up to 375 °C (with image capture at 50, 150, 270, 320, and 375 °C) and cooled to room temperature. The micrographs obtained in crossed polarizer regime are shown in Figure 8. According to the micrographs, polyesters C20 and C40 do not form the LC phase in melts up to a temperature of 375 °C. When the samples are cooled to 150 °C, crystallization occurs, which is more pronounced for the C20 sample, consistent with the results obtained by DSC and XRD.

**Figure 9:**
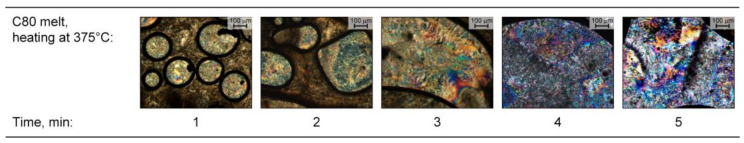
Evolution of the LC phase content over time for C80 melt.

Appearance of the LC phase for samples C60 and C80 is already observed at 270 °C, and its content significantly increases upon heating. The mesophase domains of C60 copolyester are small and surrounded by an isotropic melt. At the same time, copolyester C80 forms a mesophase throughout the entire melt volume, and the process of formation and growth of a new phase occurs relatively quickly. As shown in Figure 9, the appearance of the anisotropic melt droplets and their subsequent coalescence and spread is seen within a few minutes.

**Figure 10:**
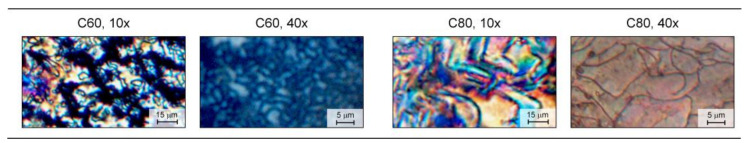
Micrographs of C60 and C80 frozen melts in crossed polarizers with 10× and 40× magnification after heating the samples to 375 °C and subsequent cooling to room temperature.

Formation of the LC phase in the C60 and C80 melts is possibly due to the following reasons: Firstly, the introduction of biphenyl fragments into PET chains makes the macromolecules more rigid, so they cannot occupy random positions in the melt but are forced to form oriented domains, i.e., elements of the LC structure. Subsequently, the domains coalesce, with the formation of a fully anisotropic melt. Secondly, the synthesis of copolyesters was carried out at a temperature of LC phase existence. Cooled C60 and C80 polymers contain LC glass and residues of the crystalline phase (Figure 8 and Figure 10). Since the solid and anisotropic phases thaw simultaneously upon heating, the enthalpy of this transition is too small to be detected by the DSC method. The isotropization temperature for C60–C80 copolymers is above 375 °C. In addition, according to TGA data, the thermal decomposition occurs above 400 °C, limiting the appearance and visualization of the isotropic phase.

Figure 10 shows micrographs of frozen C60 and C80 melts at higher magnification. Similar textures were previously characterized as nematic [36,37,41], which was to be expected for rigid rod-like macromolecules in the absence of asymmetric carbon atoms, inherent to cholesteric structure, or sequence alternation for layered (smectic) structure. The late statement was confirmed by NMR data, though for alkylene-aromatic main-chain copolyesters the smectic structure can be realized [42]. This statement is also confirmed by the XRD data: In the diffraction patterns (Figure 3a) we can see a broad signal centered at 2Θ = 20° and another broad low-intense signal at ~10°, so the cooled polymers are glassy nematic.

### 3.5. Mechanical Characteristics of Copolyesters

The values of tensile strength (*σ*), elongation at break (*ε*), and elastic modulus (*E*) for films of PET, C20–C80 and reference samples (copolyester PET/HBA, Zenite HX-8000) are given in Table 7. With increase of HBCA content, the reinforcing effect takes place, reaching the maximum for C80 sample. Values of strength and elastic modulus exceed those of PET/HBA analog and are close to fully aromatic polyesters (like Zenite HX-8000). It is important to note that such mechanical characteristics were also obtained earlier for copolyesters based on 4,4′-bibebenzoic acid, 3,4′-bibenzoic acid and EG [31], which reflects the positive role of biphenyl derivative presence in LC polyester structure.

The good mechanical characteristics of C60–C80 copolyesters, combined with increased heat resistance, can be useful for the new family of engineering plastics.

## 4. Conclusions

A series of new copolyesters of PET and 4′-hydroxy-4-biphenylcarboxylic acid containing 20–80 mol% of HBCA units was successfully synthesized by melt polycondensation. According to NMR spectroscopy, the copolymers were of random composition. Introduction of 60 mol% and more HBCA resulted in the production of copolyesters forming in melts the LC phase beginning with 270 °C. The appearance and growth of the LC phase occurred within a few minutes. The isotropization temperature of the LC melts was above the temperature of intense copolymer destruction (above 400 °C). There was a significant growth in the melt viscosity (up to two orders of magnitude) with increased biphenyl content. In addition, the formation of the LC phase was accompanied by a transition to non-Newtonian flow behavior and an increase in viscoelastic characteristics. According to XRD and polarizing microscopy data, in the solid samples after cooling LC melts this phase coexists with infusible crystallites of poly-(4′-hydroxy-4-biphenylcarboxylate). Presence of the rigid biphenyl fragments decrease segmental mobility of polymer chains that leads to increase the glass transition temperature up to 100 °C. The tensile strength and modulus for films of LC copolyesters C60 and C80 have the same or higher values compared to those of well-proven semi- and fully aromatic LC polymers. Thus, we expect these new copolyesters to be useful in obtaining heat-resistant materials with an ordered structure and, therefore, excellent mechanical characteristics.

## Figures and Tables

**Table 1:** Comonomer ratio, synthesis duration, and intrinsic viscosity of the copolyesters C20–C80.

Copolyester	Molar Ratio HBCA/(PET + HBCA), %	[*η*], dL/g	Vacuum Stage, h
C20	20	0.66	19.5
C40	40	0.70	11.0
C60	60	0.79	8.5
C80	80	2.34 ^1^	7.0
B100	100	insoluble	16.0 ^2^

^1^ logarithmic reduced viscosity in pentafluorophenol at 60 °C; ^2^ the synthesis was carried out in ditolylmethane under continuous flow of argon.

**Table 2:** Assignment of signals in the ^1^H NMR spectra of different sequences of monomeric units of C20–C80 copolymers (bold letters indicate the monomeric unit corresponding to the signals).

Sequences	Protons	Signals in the ^1^H NMR Spectrum
Aliphatic region of the spectra		
T–**E**–T, T–**E**–B	A	4.81; 4.82; 4.84
–**E**–**E**–	H K	4.14; 4.17 4.65; 4.68
–B–**E**–T	B	4.47; 4.50
Aromatic region of the spectra		
T–E–**B**–E	C	7.08 (*d*, 8.6)
B–**B**–E–T(B)	C	7.10 (*d*, 9.2); 7.11 (*d*, 8.3)
T–E–**B**–T	F	7.32 (*d*, 8.5)
B–**B**–B	F	7.34
**B**	D	7.60 (*d*, 8.2); 7.60 (*d*, 8.0); 7.71 (*d*, 8.0)
**B**	E	8.08 (*d*, 8.3); 8.12
T–E–**T**–E	G	8.14
B–E–**T**–E	G	8.16

**Table 3:** The composition of the macromolecules in copolyesters C20–C60.

	*N*_B_, %	*N*_TBE_/*N*_B_, %	*N*_BBE_/*N*_B_, %	*N*_TBT_/*N*_B_, %	*N*_BBT_/*N*_B_, %	*N*_EE_/*N*_E_, %
C20	19.7	44	13	32	11	3.1
C40	40.0	41	22	21	16	1.4
C60	59.5	29	35	22	14	3.4
C80	84.0	24		76		–

**Table 4:** Temperatures and enthalpies of phase and relaxation transitions for PET and copolyesters C20–C80.

	Glass Point, *T*_g_, °C	Melting Point, °C Enthalpy, J/g	Crystallization Point, °C Enthalpy, J/g
PET	84.3 (80.5–89.0)	257.5 (212.4–269.3) 59.5	195.9 (216.0–153.1) 48.8
C20	85.7 (80.1–91.7)	216.9 + 224.2 (195.9–235.8) 34.4	152.9 (130.2–168.8) 30.9
C40	93.9 (87.5–99.5)	197.1 (169.8–232.8) 16.9	135.5 (118.9–150.4) 14.8
C60	99.2 (90.6–107.2)	–	–
C80	100.3 (94.5–113.3)	–	–

**Table 5:** Characteristic temperatures of weight loss for PET and C20–C80 copolyesters during heating in air at a rate of 10 K/min.

Sample	Value of Weight Loss, %		
	5	10	50
	Temperature at Which Weight Loss Is Observed, °C		
PET	391	411	437
C20	373	403	430
C40	414	426	470
C60	417	431	486
C80	417	435	531

**Table 6:** The values of the melt viscosity of C20–C80 copolyesters measured in the stationary shear (*η*) and oscillation (|η*|) modes at equal shear rates ($\dot{\gamma }$) and frequencies (ω) at 270 °C.

Sample	$\dot{\gamma }=\omega =1\;s^{-1}$		$\dot{\gamma }=\omega =25\;s^{-1}$	
	*η*, Pa·s	|η*|, Pa·s	*η*, Pa·s	|η*|, Pa·s
C20	12	11	11	10
C40	46	53	41	49
C60	111	183	62	102
C80	486	747	184	306

**Table 7:** Mechanical characteristics of C20–C80 copolyester films in comparison with PET, and other similar previously described copolyesters. The average film thickness was 0.17 ± 0.03 mm for all samples.

Sample	Elongation at Break, *ε* (%)	Tensile Strength, *σ* (MPa)	Elastic Modulus, *E* (MPa)
PET	450 ± 14	31 ± 1	960 ± 160
C20	65 ± 10	29 ± 4	910 ± 90
C40	21 ± 7	30 ± 3	900 ± 60
C60	8 ± 2	38 ± 3	930 ± 50
C80	6 ± 1	54 ± 4	1600 ± 90
PET–HBA (40/60) ^1^	19 ± 2	44 ± 8	1100 ± 110
Zenite HX-8000 ^2^	22 ± 2	61 ± 9	1100 ± 140

^1^ the analog of Rodrun LC-polymer; ^2^ the commercial product.

## Data Availability

The data presented in this study are available on request from the corresponding author.
